# Medical Specialities

**Published:** 1862-11

**Authors:** 


### Editorial.

### MEDICAL SPECIALTIES.

THIS is rather a delicate subject, nevertheless we have somewhat to say, and in the first place, we think it an evidence of decided progress in the treatment of diseases, that divisions and definite departments of medical practice are being made, and each receiving the undivided and concentrated attention and effort of those devoted to the respective specialties.

The advantages of such divisions are many and obvious. There are certain fundamental principles and laws, which all who attempt the treatment of disease of any organ, or kind, should fully understand ; for instance, the principles of anatomy physiology, pathology, therapeutics, and organic chemistry ; these should constitute the basis of every practitioner's attainments, it matters not to what particular part he may direct his attention. And the medical student who has completed the ordinary course of study in these general subjects, is then just ready to begin to learn his special duties. He is no more fit to take charge of, and treat grave diseases, than the novice who had made all his attainments by reading and lectures, would be to take charge of a steam ship's engine, when all broken and out of gear, and the ship about to founder.

The matter of medical specialties has been very very much discountenanced by most general practitioners, and not without some very good reasons, as things have existed, two or three of which we may enumerate. And first, it was supposed by specialists to be unnecessary to give any attention to the general branches we have referred to, or to look farther than the mere point or organ which was to be the field of their operation and treatment : hence, a great many very ignorant persons have attempted to practice some particular department, without the attainments which would entitle them to any recognition by the medical profession ; yet such persons are oftentimes warmly sustained by the people, and perhaps sometimes as much because they are not thus recognized, as from any supposed merit.

Again, specialties in medical practice have constituted a luxuriant field for quackery. Quackery and ignorance are not necessarily connected; the ignorant man may act from the purest motives and accomplish what he does in the best manner, without any attempt at self-exaltation; the quack, on the other hand, may be one of great attainments, but he prostitutes every thing to a base and selfish purpose: self praise and exaltation being the chief object of all he does. The people are strongly inclined to countenance and encourage those who practice specialties; and upon a very correct principle, viz.: that one who gives all his attention to one thing, should do it much better than he who tries to do very many diverse things.

In all this a change is taking place. Many now who practice only in particular departments are men of great attainments, not only in that with which they are immediately concerned, but in all collaterals, and indeed in many things that can not be thus termed.

He who pushes his investigations all the while in one direction will penetrate much deeper, and more perfectly, into the mysteries of natuie, than he who attempts to operate in a much larger field: hence, the good specialist, if he is a thorough student, will become better acquainted with the principles of physiology and pathology, than the general practitioner; indeed, in the science of medicine, it is this class of men who, to a very great extent, make the discoveries and improvements.

This division of medical practice is becoming more and more extended, and more definite. No intelligent person would think of applying to the general practitioner of medicine for treatment of diseased teeth; the intelligent physician himself always applies to the dentist. No one will apply to the general practitioner for the treatment of diseased eyes or ears: and why? because some one else can do it better; neither will any one apply to the general physician for a difficult and dangerous surgical operation; but he will go at once to the surgeon, who is thoroughly skilled in his department, or at least has that skill which springs from an entire devotedness to his subject.

Judging from the results thus obtained by devotion to specialties, we are disposed to believe it would be vastly advantageous to mankind if such a division should run throughout medical practice; we think the tendency is in that direction; and soon may it come. T.

